# Analysis of genetic diversity and population structure of some Ethiopian barley (*Hordeum vulgare* L.) accessions using SSR markers

**DOI:** 10.1371/journal.pone.0305945

**Published:** 2024-06-25

**Authors:** Alemayehu Zewodu, Wassu Mohammed, Eleni Shiferaw

**Affiliations:** 1 Department of Crop and Horticulture Biodiversity Research, Ethiopian Biodiversity Institute, Addis Ababa, Ethiopia; 2 Department of Plant Science, Haramaya University, Haramaya, Ethiopia; Arish university, Faculty of agricultural and environmental sciences, EGYPT

## Abstract

Understanding the genetic diversity of existing genetic resources at the DNA level is an effective approach for germplasm conservation and utilization in breeding programs. However, the patterns of genetic diversity and population structure remain poorly characterized, making germplasm conservation and breeding efforts difficult to succeed. Thus, this study is aimed to evaluate the genetic diversity and population structure of 49 barley accessions collected from different geographic origins in Ethiopia. Twelve SSR markers were used to analyze all accessions and a total of 61 alleles were found, with a mean of 5.08 alleles per locus. The analysis pointed out the existence of moderate to high values of polymorphic information content ranging from 0.39 to 0.91 and the mean Shannon diversity index(I) was 1.25, indicating that they were highly informative markers. The highest Euclidean distance (1.32) was computed between accession 9950 and two accessions (247011 and 9949), while the lowest Euclidean distance (0.00) was estimated between accessions 243191 and 243192. The result of molecular variance analysis revealed that the highest variation was found among accessions (47) relative to within accessions (44) and among geographic origins (9). Cluster analysis grouped the 49 barley accessions into three major clusters regardless of their geographic origin which could be due to the presence of considerable gene flow (2.72). The result of the STRUCTURE analysis was consistent with neighbor-joining clustering and principal coordinate analysis. Generally, this study concluded that the variation among accessions was more important than the difference in geographical regions to develop an appropriate conservation strategy and for parental selection to use in breeding programs. This information will be helpful for barley conservation and breeding, and it may speed up the development of new competing barley varieties.

## Introduction

Barley (*Hordeum vulgare* L.) belongs to the grass family Poaceae or Gramineae and ranks fourth in global production among cereal crops next to wheat, rice and maize [1]. It is a self-pollinated diploid (2n = 2x = 14) crop species with a relatively large genome size of 5.1 Gbp [2]. Barley is believed to have originated from its closest wild relative, *Hordeum spontaneum* in the Near East’s Fertile Crescent area [3]. Ethiopia is regarded as a center of barley diversity [4], with a significant level of morphological variation between landraces resulting from adaptation to diverse climatic conditions and soil types [5]. Barley is an early maturing cereal crop with high-yielding potential in a wide range of environments, including marginal areas that are not favorable for the production of other cereals. It is predominantly cultivated at altitudes between 2400 and 3400 m. a. s. l in Ethiopia [6].

Globally, barley is grown in more than 100 countries: Europe has been the leading country in the world in barley production followed by North and Central America and Asia for the last four decades(1). It is a significantly important grain throughout the world and is commonly used for animal feed, the production of malt and human consumption [7]. Barley has been in cultivation for at least the past 5000 years in Ethiopia. The first Ethiopians to have ever grown barley are believed to be the Agew people, in approximately 3000 BC [8]. Currently, barley is the fifth most important cereal crop in Ethiopia and accounts for about 7.74% of the total national cereal production, with an average yield of 2.53 ton per hectar [9]. In the highlands of Ethiopia, barley is a staple food and its straw serves as animal feed [10, 11].

Knowledge concerning the amount of genetic variation, population structure and genetic relationships between germplasms is an important consideration for designing appropriate conservation strategies and utilization of germplasm collections [12, 13]. Genetic diversity is the amount of genetic variation among individuals within a species and it is indispensable for germplasm conservation strategies. Genetic diversity provides the raw material for adaptation to biotic and abiotic stresses [14, 15], enabling the introgression of desirable traits into breeding programs and increasing the genetic gain in breeding programs [16, 17]. Evaluation of the genetic diversity in crop species can be performed by different methods such as pedigree records, morphological traits, biochemical and molecular markers [18]. Numerous studies have successfully used classical markers to identify genetic variation, in Ethiopian barley accessions from different geographic origins, such as those based on morphological markers [19–22] and biochemical markers [23–25]. However, those markers are limited in number, they detect low levels of polymorphism and are influenced by different plant growth stages and environmental factors [26, 27]. To address this limitation and obtain reliable information, morphological characterization should be supported with molecular markers.

Previous studies explored the genetic diversity of Ethiopian barley genotypes useing various molecular markers such as Restriction Fragment Length Polymorphisms (RFLPs) [28, 29] and Amplified fragment length polymorphisms (AFLPs [30]. However, simple sequence repeat markers are a marker of choice due to their high reproducibility, greater genome abundance, codominant inheritance, informativeness and simple detection system [31, 32]. Simple sequence repeat markers are short tandem repeats with higher levels of polymorphism and are widely distributed throughout the genome [33, 34]. It is an ideal marker for assessing genetic diversity, identifying appropriate parental lines for breeding, and managing germplasm collections [35]. Several genetic diversity studies were performed in different germplasm accessions in Ethiopia using SSR [13, 36–39]. However, these and other studies generated information from a small proportion of the available germplasm.

Currently, Ethiopian Biodiversity Institute is conserving many barley accessions from various parts of the country. Despite many collections, the level and distribution of genetic diversity are still poorly understood, which limits attempts to use this diversity for crop improvement and adaptation to fluctuating environmental conditions. In addition, the population structure, including the presence of distinct subpopulations, admixture patterns and gene flow, has not been adequately characterized, hindering the adoption of targeted breeding approaches and germplasm conservation efforts. Therefore, the current study aimed to examine the genetic diversity and population structure of barley accessions collected from various geographic origins.

## Materials and methods

### Plant materials

**Fig 1:**
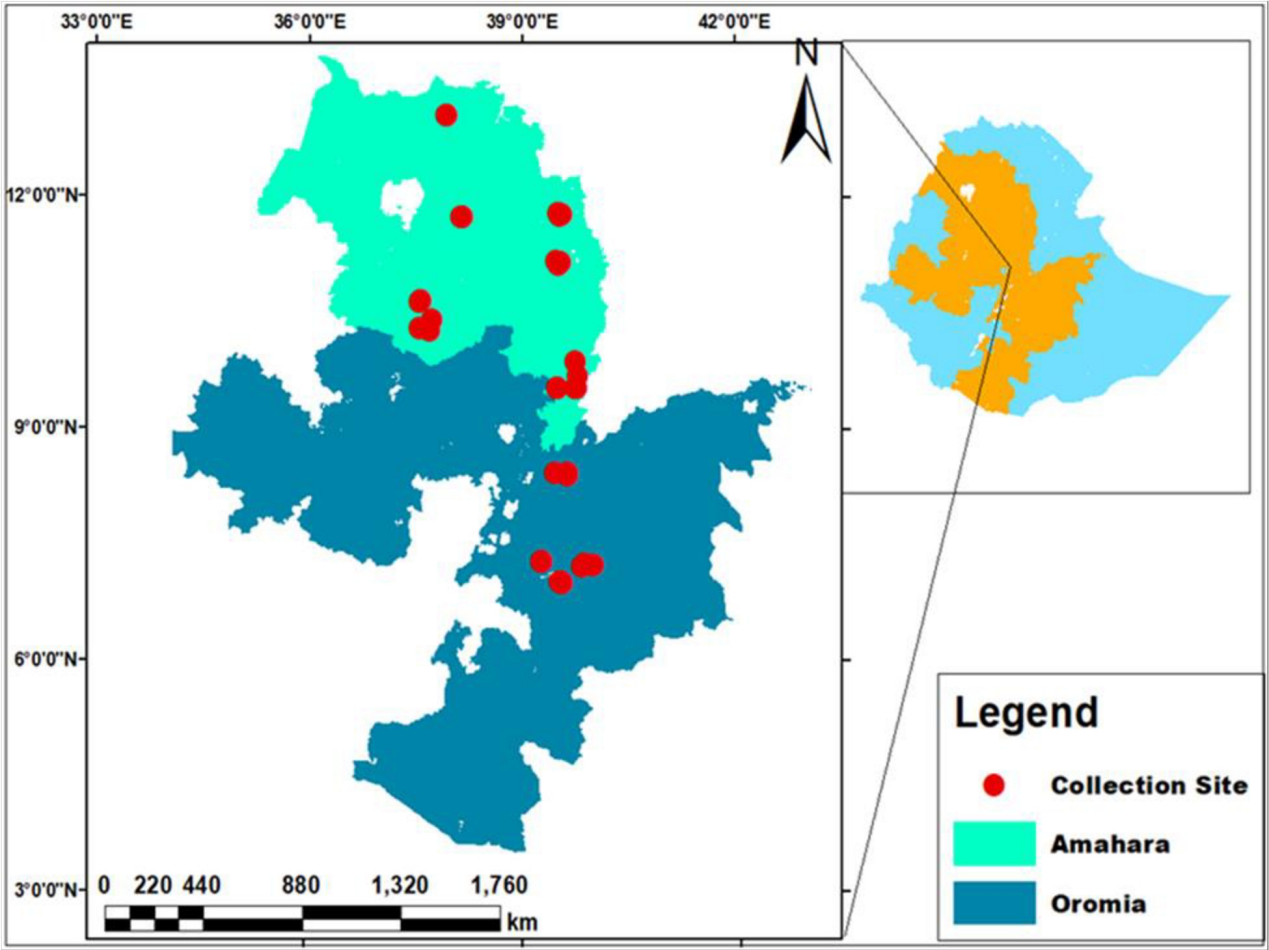
Map of Ethiopia showing the collection regions of barley accessions used in this study.

A total of 49 barley accessions collected from parts of Amhara and Oromiya regions by Ethiopian Biodiversity Institute were used for this study (Fig 1). The accessions were selected based on the passport data from different geographical locations conserved in genebank (S1 Table). The study area map was constructed using the coordinates of the collection sites in ArcGIS software.

### DNA extraction and polymerase chain reaction(PCR) amplification

The leaf tissues were collected from 10 randomly selected plants from each accession and preserved in silica gel until DNA extraction. DNA was extracted based on a modified cetyltrimethyl ammonium bromide (CTAB) protocol [40]. Silicagel dried leaf samples were crushed into fine powder using a mortar and pestle in 750 μL CTAB buffer (0.35 M sorbitol, 0.1 M Tris-HCl (pH 7.6), 0.005 M Ethylenediaminetetraacetic acid (EDTA), 0.2 M Tris-HCl (pH 8.0), 0.05 M EDTA, 2% CTAB and 2M Sodium chloride (NaCl)) in a 2 ml microcentrifuge tube. DNA was quantified using a nanodrop and the quality was checked using 1% agarose gel electrophoresis. From each accession, DNA samples from 10 individual plants were bulked for subsequent simple sequence repeat (SSR) analysis [41]. The working DNA samples were diluted to a concentration of 30 ng/μL and stored at -20°C. Fourteen SSR markers, two from each of the seven linkage groups with known map locations, were selected from published literature [42–45] and used in this study (Table 1).

**Table 1:** List and description of SSR primers used for this study.

SSR Marker	Primer sequence (5’ →3’)	Repeat motif	Chromosome location	Size(bp)	Annealing T(°C)	Reference
bmag0211	F: ATTCATCGATCTTGTATTAGTCC	(CT)16	1	173–204	52	[43]
	R: ACATCATGTCGATCAAAGC					
WMC1E8	F: TCATTCGTTGCAGATACACCAC	(AC)24	1	198–217	57	[44]
	R: TCAATGCCCTTGTTTCTGACCT					
Bmag0711	F: GGAGAGTCACATATCAAGGAC	(GA)21	2	193–247	55.6	[43]
	R: CTTCGTTGCTTCTCTACCTT					
Ebmac0415	F: GAAACCCATCATAGCAGC	(AC)17	2	210–270	55	[42]
	R: AAACAGCAGCAAGAGGAG					
GBM1405	F: TACACGCACTGAAAAGACGG	(CGCA)5	3	290–310	55	[43]
	R: CTCGCTGCTGAGTTTGTCTG					
GBM1413	F: GGGTGATTTCCCAGGTTTTT	(TCATA)6	3	167–184	55.5	[45]
	R: CTCGCTGCTGAGTTTGTCTG					
Bmag0808	F: TCATAGACTACGACGAAGATG	(GA)16	4	160–174	55	[43]
	R: TCTTTGGATGTGTGTTTACTG					
Bmac0096	F: GCTATGGCGTACTATGTATGGTTG	(AT)6(AC)18	4	155–180	57	[43]
	R: TCACGATGAGGTATGATCAAAGA					
GBM1176	F: TATACATCAGCGGGCCTTTT	(AT)8	5	272–362	53	[45]
	R: CTCCAACCCCTCGCAAAGAGTC					
Scssr02306	F: TGCCTTGTTTATGTAATATCTTGTG	(AT)6 (CA)7	5	140–163	55	[43]
	R: GGCGTAAATAAGAGTGTCTTCAG					
Bmac0018	F: GTCCTTTACGCATGAACCGT	(AC)11	6	145–163	55	[45]
	R: ACATACGCCAGACTCGTGTG					
Bmac0316	F: ATGGTAGAGGTCCCAACTG	(AC)19	6	135–141	55	[42]
	R: ATCACTGCTGTGCCTAGC					
GBM1419	F: CGTCACGCCACTCACCTC	(CTCAT)5	7	92–105	55	[43]
	R: CTTGAAGTCGGAACCCATGT					
GBM1464	F: ATAGCCGTGCTCTTGCTCAT	(CAG)8(CAG)5	7	168–211	52	[43]
	R: CAAGACCACCATTTGCATTG					

F = Forward, R = Reverse

Polymerase chain reaction (PCR) was carried out in a total volume of 15 μL consisting of 30 ng/μL genomic DNA, 1.5 μL of 10x reaction buffer, 1.5 mM MgCl_2,_ 0.2 mM dNTPs, 0.4 μM each of the forward and reverse primers (Invitrogen, Thermo Fisher, Inc.), 0.05u/μL DNA polymerase (Solis bioDyne, Estonia) in Hybaid PCR express Thermal cycler. Target DNA fragments were amplified using the following PCR profile: an initial denaturing step of 5 min at 94°C followed by 35 cycles with denaturation at 94°C for 30 s, annealing at optimum temperature for 30 s and extension at 72°C for 45 min. After 35 cycles, a final extension step was performed at 72°C for 10 minutes. PCR products were resolved on a 6% polyacrylamide gel in 0.5x TBE buffer with a 6x DNA loading dye. Vertical electrophoresis was carried out for two hours and 30 minutes at 100 V and gels were stained with silver staining based on the protocol developed by [46] and documented using a digital camera. Fragment sizes of each locus were estimated by comparison with the standard 100 bp DNA ladder (Solis bioDyne, Estonia). The gel images were documented and the band sizes were determined using UVITEC image analysis software (UVITEC, Cambridge, UK).

### Scoring data and statistical analysis

The individual amplified DNA fragment sizes in each SSR marker were recorded using UVITEC software. The genotypic data were used to analyze locus-based diversity indices such as the number of alleles (Na), effective number of alleles (Ne), percentage of polymorphic loci, observed heterozygosity (Ho), expected heterozygosity (He), Shannon information index (I) and gene flow (Nm) using GenAlEx software v 6.5 [47]. To check the informativeness of the SSR markers used in this study, polymorphic information content (PIC) was analyzed using Power-Marker 3.25 [48]. To partition total genetic variation within and among populations; estimates of genetic differentiation were computed by analysis of molecular variance (AMOVA) using GenAlEx 6.5 [47]. Principal coordinate analysis (PCoA) was conducted from the distance matrix of each accession using GenAlEx software.

The dendrogram tree was constructed by the unweighted pair-group method with arithmetic averages (UPGMA) based on Euclidian distance using PowerMarker 3.25 [48] and the tree was visualized using Molecular Evolutionary Genetic Analysis (MEGA 6) [49]. The Bayesian model-based software STRUCTURE 2.3.4 [50] was used to infer the population structure for the sampled accessions. The analysis of Population structure was performed 10 times for each K value (K = 1 to 10) using a burn-in period of 50,000 and 100,000 Markov chain Monte Carlo (MCMC) iterations. The optimal K value among K groups was determined based on [51] using STRUCTURE HARVESTER [52]. The result files obtained from STRUCTURE HARVESTER were also analyzed by CLUMPP software [53] to align the clusters across replicates and to display clusters in each K drawn as colored box plots.

## Results

### Genetic diversity parameters from SSR markers

Out of the 14 primers that were used in this study, 12 microsatellite primers were polymorphic, while the two were monomorphic. A total of 61 polymorphic alleles were amplified with an average of 5.08 alleles per locus (Table 2). The major allele frequency varied from 0.15 (GBM1176 and GBM1464) to 0.73 (Bmac0018) with an average of 0.35. The mean number of effective alleles (Ne) was 3.6 ranging from 1.67 (for Bmac0018) to 6.07 (for GBM1176). The values of Shannon’s information index (I) varied from 0.61 for locus Bmac0018 to 1.82 for locus GBM1176 with a mean value of 1.25. With a mean value of 0.64, the expected heterozygosity ranged from 0.39 for locus Bmac0018 to 0.80 for locus GBM1176 (Table 2). The average number of fixation index (Fst) was 0.13 with numbers varying from 0.04 (WMC1E8) to 0.23 (GBM1419). The average number of gene flows (Nm) in the entire population ranged from 0.91 (GBM1419) to 6.16 (WMC1E8), with an average of 2.13 among all loci. The mean values polymorphic information content (PIC) of the current study was 0.73, with values ranging from 0.39 (Bmac0018) to 0.91 (GBM1464) (Table 2).

**Table 2:** Genetic diversity parameters based on 12 SSR markers among 49 barley accessions.

Locus	MAF	Na	Ne	I	He	Ho	Fst	Nm	PIC
bmag0211	0.35	4	3.08	1.16	0.63	0.00	0.17	1.23	0.77
WMC1E8	0.43	5	2.78	1.17	0.64	0.80	0.04	6.16	0.60
bmag0711	0.37	5	2.48	1.11	0.58	0.41	0.18	1.12	0.69
Ebmac0415	0.24	7	5.29	1.65	0.77	0.97	0.07	3.13	0.84
GBM1413	0.33	4	3.20	1.11	0.63	0.00	0.15	1.40	0.75
GBM1419	0.45	3	2.05	0.77	0.49	0.00	0.23	0.91	0.59
bmag0808	0.33	4	2.80	1.06	0.59	0.00	0.21	0.95	0.70
GBM1176	0.15	8	6.07	1.82	0.80	0.94	0.10	2.24	0.89
Scssr02306	0.35	4	3.04	1.15	0.66	0.00	0.10	2.32	0.72
Bmac0018	0.73	3	1.67	0.61	0.39	0.00	0.08	2.78	0.39
Bmac0316	0.16	7	5.47	1.66	0.76	1.00	0.13	1.74	0.87
GBM1464	0.15	7	5.25	1.72	0.78	0.11	0.14	1.57	0.91
Total	4.04	61	43.18	14.99	7.72	4.23	1.60	25.55	8.72
Mean	0.35	5.08	3.6	1.25	0.64	0.35	0.13	2.13	0.73
SE	-	0.37	0.3	0.07	0.02	0.02	0.02	0.42	-

MAF = Major allele frequency, Na = Number of alleles detected per locus, Ne = Number of effective alleles, I = Shannon’s information index, Ho = Observed heterozygosity, He = Expected heterozygosity, Fst = Fixation index, Nm = Gene flow, PIC = Polymorphic information content and SE = Standard error.

### Allelic diversity among geographic origins

**Fig 2:**
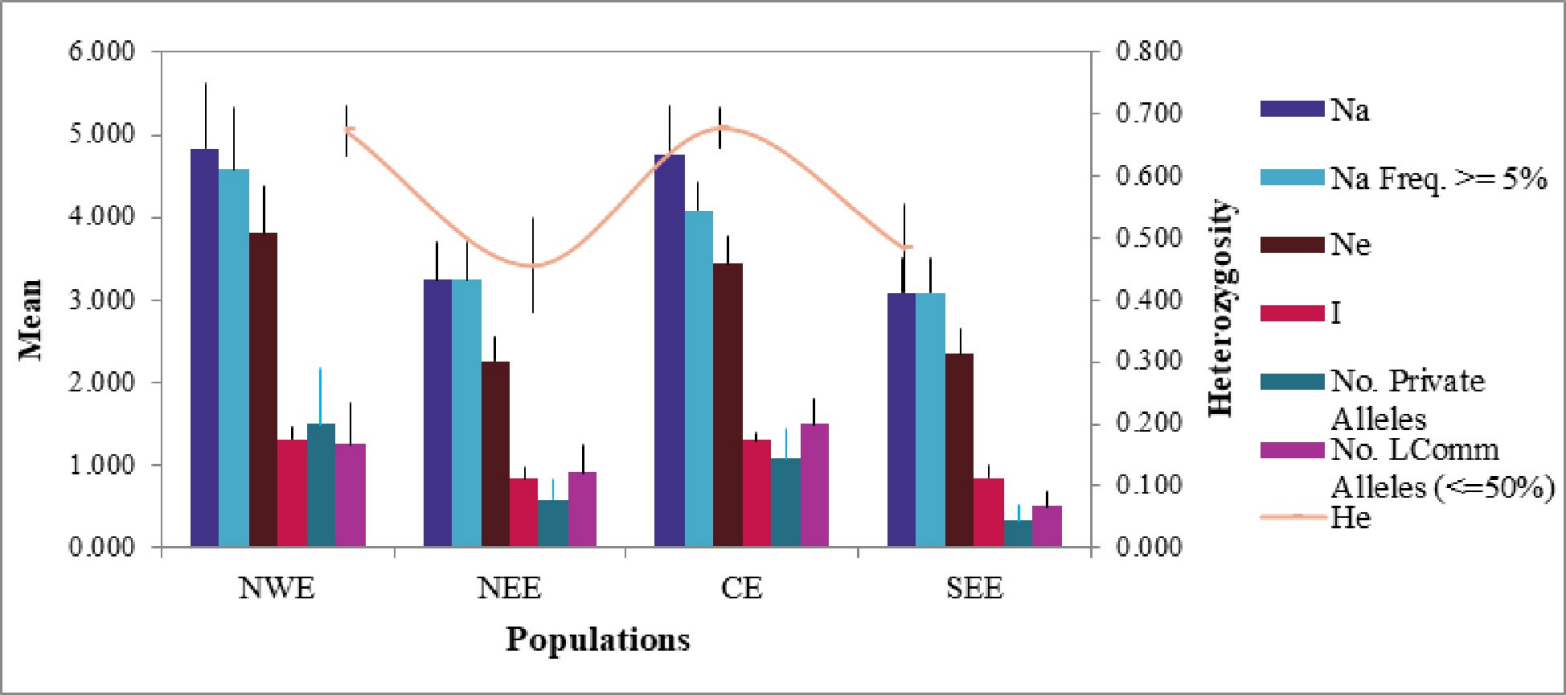
Allelic patterns of 49 Ethiopian barley accessions across four geographical regions. NWE = Northwest Ethiopia, NEE = Northeast Ethiopia, CE = Central Ethiopia, SEE = Southeast Ethiopia.

The genetic diversity of 49 barley accessions across four geographic origins is summarized in Table 3. Accessions from central Ethiopia (6.25) had the highest number of different alleles, followed by northwest Ethiopia (5.58), and accession from southeast Ethiopia (3.17), showed the lowest number of different alleles followed by northeast Ethiopia (3.42) (Table 3 and Fig 2). The number of effective alleles ranged between 2.59 (northeast Ethiopia) and 4.76 (central Ethiopia). The observed heterozygosity (Ho) in geographic origin varied from 0.30 (northeast Ethiopia) to 0.39 (central Ethiopia), whereas the expected heterozygosity (He) ranged from 0.56 (northeast Ethiopia) to 0.73 (central Ethiopia). with a mean of 0.50, the estimated fixation index (F) varied from 0.45 (southeast Ethiopia) to 0.55 (northwest Ethiopia). The percentage of polymorphic loci (% P) across all populations was 100% (Table 3).

**Table 3:** Summary of genetic diversity in barley accessions from four geographic origins.

Population	N	Na	Ne	NPA	I	Ho	He	%P
Northwest Ethiopia	16	5.58	4.44	0.41	1.47	0.35	0.71	100
Northeast Ethiopia	8	3.42	2.59	0.20	0.99	0.30	0.56	100
Central Ethiopia	17	6.25	4.76	0.32	1.55	0.39	0.73	100
Southeast Ethiopia	8	3.17	2.60	0.17	0.99	0.36	0.58	100
Mean	12.25	4.61	3.60	0.28	1.25	0.35	0.65	100

N = No. of samples, Na = No. of different alleles, Ne = No. of effective alleles, NPA = No. of private alleles, Ho = observed heterozygosity, He = expected heterozygosity, I = shannon’s information index, %p = Percentage of Polymorphic Loci

### Genetic distance

The genetic distances for all possible pairs of 49 barley accessions are presented in S2 Table. The genetic distance of 1176 pairs of accessions ranged from 0.00 to 1.32, with a mean, standard deviation and coefficient of variation of 0.94, 0.20 and 21.68%, respectively (Table 4). The highest Euclidean distance (1.32) was computed between the accession 9950 and two accessions (247011 and 9949) followed by 1.31 Euclidean distance estimated between accession 243229 and four accessions (247011, 243214, 243215 and 243216) and Euclidean distance of 1.29 between 232222 and 237011. The lowest Euclidean distance (0.00) was estimated between 243191 and 243192 followed by (0.12) computed between 243191 and 243193 and between 243192 and 243193 (S2 Table). Generally, 4.10% pairs of accessions had genetic distances <0.49, 50.85% pairs of accessions had genetic distances in between 0.50–0.99 and 45.10% pairs of accessions had genetic distance between 1.00 and 1.32 (S2 Table). According to the estimated mean Euclidean distance, accession 237011 (1.12) followed by 8525 (1.05) and 4427 (1.04) were the most distant accessions from the other accessions, whereas 243600 (0.84) followed by 243599, 243572 and 243286 (0.86) were found to be the closest to the other accessions (Table 4).

**Table 4:** Range, mean, standard deviation and coefficient of variation of Eucludian distance based on 12 SSR markers.

Accen.	Min	Max	Mean	SD	CV(%)	Accen.	Min	Max	Mean	SD	CV(%)
212737	0.33	1.31	0.97	0.18	18.64	243232	0.42	1.28	0.98	0.18	18.65
232219	0.24	1.21	0.93	0.16	17.35	243286	0.35	1.12	0.86	0.18	21.10
232220	0.24	1.24	0.94	0.15	16.49	243287	0.29	1.19	0.89	0.19	21.09
232221	0.16	1.22	0.91	0.17	19.25	243288	0.29	1.21	0.94	0.17	18.01
232222	0.16	1.29	0.96	0.19	19.97	243289	0.68	1.26	0.97	0.14	14.47
235066	0.24	1.27	0.96	0.21	21.42	243568	0.44	1.24	0.92	0.19	20.52
235072	0.24	1.24	0.91	0.22	24.69	243571	0.46	1.24	0.98	0.18	18.29
235073	0.28	1.24	0.88	0.23	25.78	243572	0.41	1.24	0.86	0.21	24.55
235074	0.28	1.24	0.90	0.22	24.80	243597	0.46	1.24	0.91	0.19	20.29
235075	0.28	1.30	0.94	0.22	23.84	243598	0.20	1.18	0.88	0.21	23.36
237002	0.30	1.23	0.87	0.22	25.07	243599	0.12	1.18	0.86	0.23	27.02
237003	0.30	1.24	0.87	0.22	25.03	243600	0.12	1.18	0.84	0.23	26.83
237004	0.32	1.25	0.90	0.21	23.62	4366	0.26	1.19	0.94	0.18	19.04
237011	0.53	1.32	1.12	0.17	15.02	4423	0.26	1.17	0.97	0.17	17.91
243191	0.00	1.25	0.88	0.25	28.98	4425	0.34	1.13	0.92	0.17	18.68
243192	0.00	1.25	0.88	0.25	28.98	4426	0.24	1.24	1.00	0.20	20.08
243193	0.12	1.25	0.88	0.25	28.96	4427	0.24	1.24	1.04	0.20	19.31
243195	0.18	1.27	0.92	0.22	23.78	8525	0.50	1.27	1.05	0.18	16.66
243213	0.41	1.28	0.97	0.18	18.21	8526	0.50	1.23	0.97	0.18	18.74
243214	0.41	1.31	0.98	0.22	21.99	8556	0.57	1.30	1.03	0.18	17.35
243215	0.18	1.31	0.88	0.18	20.60	8557	0.50	1.24	0.99	0.14	14.04
243216	0.41	1.31	0.98	0.20	20.55	8558	0.50	1.29	0.95	0.17	18.24
243229	0.18	1.31	0.88	0.18	20.35	9949	0.24	1.32	1.00	0.18	18.29
243230	0.50	1.15	0.94	0.18	19.41	9950	0.24	1.32	0.98	0.18	18.62
243231	0.49	1.25	1.01	0.16	15.67	overall	0	1.32	0.94	0.20	21.68

Accen = Accessions, Min = Minimum, Max = Maximum, SD = Standared deviation, CV = Coefficient of variation

### Analysis of molecular variance and genetic differentiation

Analysis of molecular variance (AMOVA) was conducted to partition the observed variation of accessions into sources of genetic differentiation. The result revealed that 44% of the total variance was attributed to genetic variation within accessions and the variation among accessions within groups (47%). Only 9% of the total variance was associated with differentiation among the geographic regions (Table 5). Gene flow (Nm) between populations of four geographical regions ranged from 1.15 (between Northeast and Southeast Ethiopia) to 6.99 (between Central and Northwest Ethiopia), with an average gene flow of 4.07 among populations of regions which was also found to be high. The barley populations from northeast Ethiopia and southeast Ethiopia had the highest genetic differentiation (Fst) value (0.18), whereas barley populations from northwest Ethiopia and central Ethiopia had the lowest Fst value (0.04) (Table 6).

**Table 5:** Analysis of molecular variance among and within the population of barley accession using 12 SSR markers.

Source of variation	Df	SS	MS	Est.Var.	Explained Var. in %	Fst	Nm
Among geographic regions	3	47.9	16	0.4	9	0.084	2.72
Among accessions	45	296	6.6	2.2	47		
Within accessions	49	103	2.1	2.1	44		
Total	97	446.9	-	4.7	100		

Df = Degree of freedom, SS = Sum of squares, MS = Mean square, Est. Var = Estimated variance, Explained Var. in % = Explained variance in percent, Fst = Genetic differentiation, Nm = Estimates of gene flow.

**Table 6:** Pairwise population differentiation above the diagonal and gene flow below the diagonal among barley populations from various geographic origins.

Population	Northwest Ethiopia	Central Ethiopia	Northeast Ethiopia	Southeast Ethiopia
Northwest Ethiopia	--	0.040	0.090	0.098
Central Ethiopia	6.987	--	0.105	0.103
Northeast Ethiopia	2.70	2.142	--	0.178
Southeast Ethiopia	2.311	2.177	1.154	--

### Clustering

**Fig 3:**
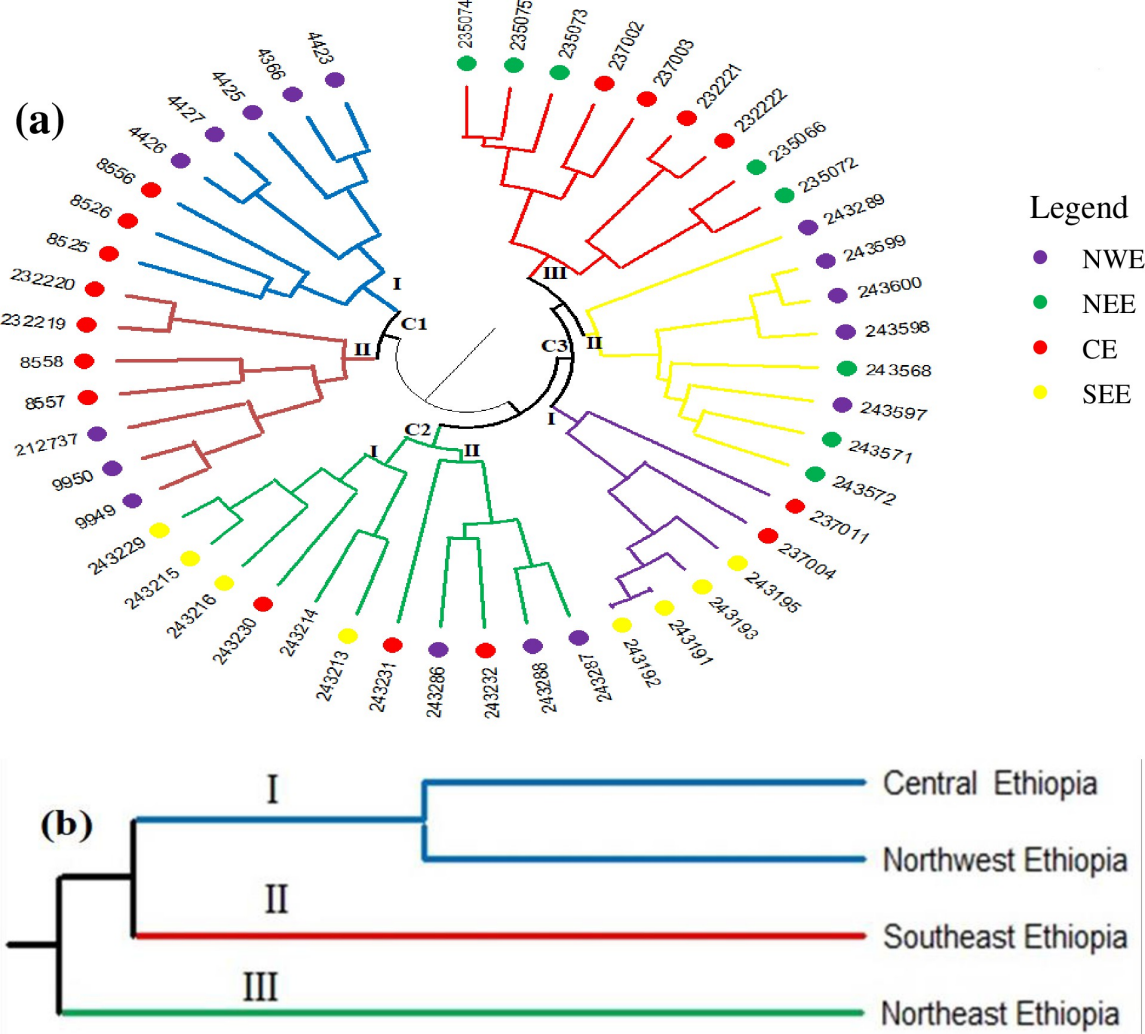
UPGMA dendrogram showing the genetic relationships among 49 barley accessions(a) and among the four populations (b) using 12 polymorphic SSR markers. NWE = Northwest Ethiopia; NEE = Northeast Ethiopia; CE = Central Ethiopia; SEE = Southeast Ethiopia.

The 49 accessions collected from four different geographic regions were divided into three clusters, with cluster I, II, and III consisting of 15 (30.61%), 11 (22.45%), and 23 (46.94%) accessions, respectively (Fig 3A). Cluster I divided into two subgroups that consisted of eight (53.33%) accessions from northwestern Ethiopia and seven (46.66%) accessions from central Ethiopia. Cluster II was divided into two subgroups of which four accessions each were collected from southeastern and central Ethiopia while three accessions were collected from northwestern Ethiopia. Cluster III was divided into three subgroups of which subgroup I was constructed by six accessions, four and two accessions collected from southeastern and central Ethiopia, respectively. Subgroup II consisted of five and three accessions collected from northwestern and northeastern Ethiopia, respectively. Subgroup III contained five and four accessions collected from northeastern and central Ethiopia, respectively (Fig 3A). The UPGMA dendrogram exhibited the presence of genetic relationships accross the four barley populations and grouped them into three clusters, of which Cluster I consisted of accessions from central constructed solitary Clusters II and III, respectively (Fig 3B).

### Principal coordinates and population structure analysis

**Fig 4:**
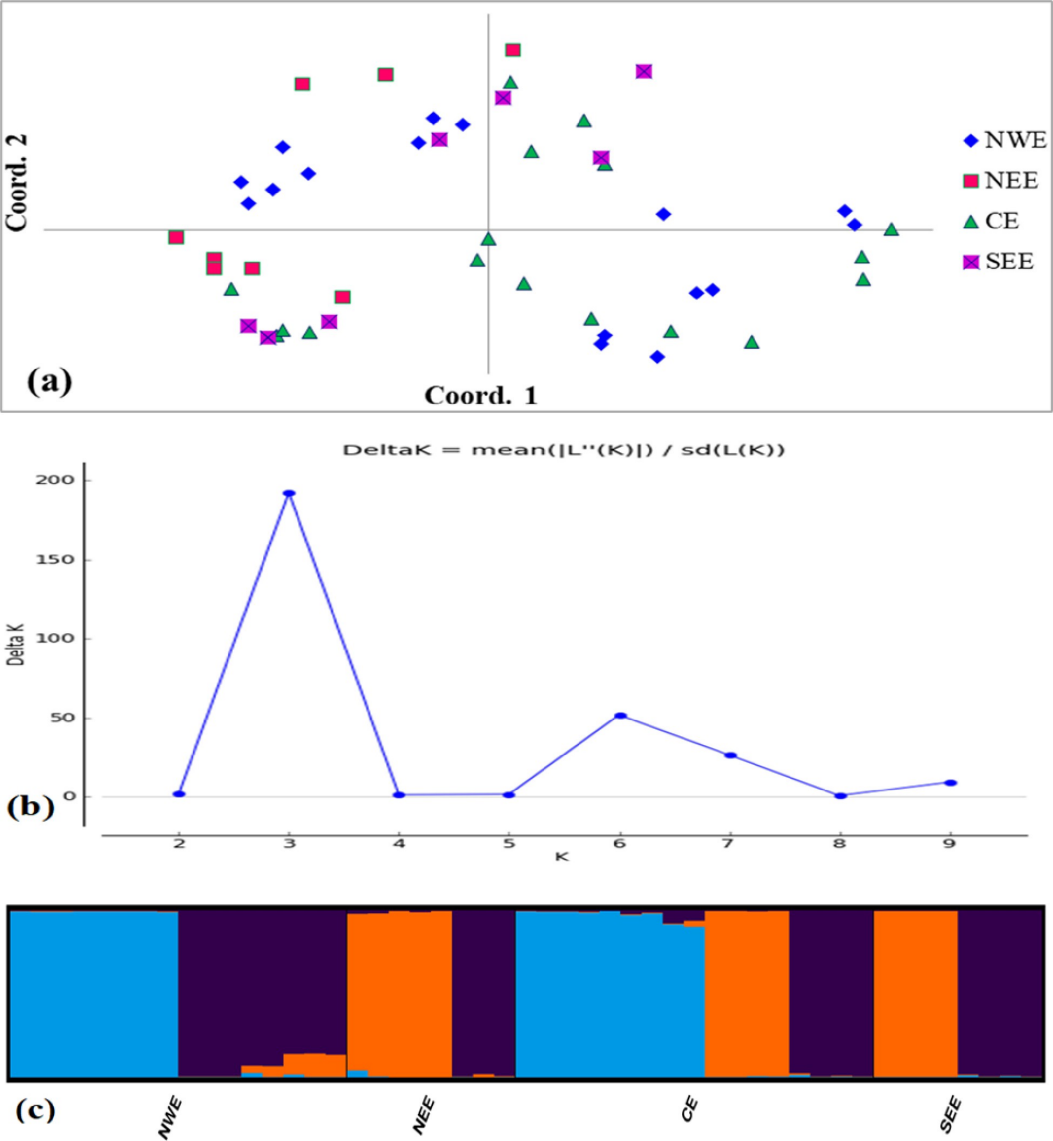
(a) Principal coordinate analysis of 49 barley accessions; (b) Delta k values for determining the optimal number of subpopulations; (c) Population structure of 49 barley accessions representing four geographic regions in Ethiopia at k = 3. NWE = Northwest Ethiopia, NEE = Northeast Ethiopia, CE = Central Ethiopia, SEE = Southeast Ethiopia.

The result of principal coordinate analysis (PCoA) showed the wide distribution of accessions across four quadrants regardless of their geographic origins (Fig 4A). The first three coordinates explained 33.14% of the total variation, with 13.2%, 11.2% and 8.7% contributions for dimensions 1, 2, and 3, respectively. The populations of central Ethiopia showed admixture with populations of Northwest Ethiopia and Southeast Ethiopia. At the same time, there was also some overlap between the populations of Northeast Ethiopia and those of Northwest Ethiopia. The population structure of 49 barley accessions was determined using structure 2.3.4, and the result of the structure harvester revealed that the largest peak was found at k = 3 indicating the presence of three subpopulations (Fig 4B and 4C).

## Discussion

In this study, the genetic diversity and population structure of 49 barley accessions were investigated using 12 polymorphic SSR markers to provide information for designing a fruitful breeding program and germplasm conservation strategy. The 12 polymorphic SSR markers yielded an average of 5.08 alleles per locus, which was lower than the 9.76 alleles per locus reported by [13] and 9.4 per locus found by [39], but higher than the 2.76 per locus reported by [54]. The observed variation in the different studies might be due to the difference in the origin of the genotypes and the number of genotyped accessions with different genetic constituents and the nature and polymorphism level of markers used in the different studies.

The loci such as bmag0211, bmag0711, GBM1413, GBM1419, bmag0808, Scssr02306, Bmac0018 and GBM1464 showed lower observed heterozygosity than expected heterozygosity. In addition, they had high fixation index, which indicates high level of inbreeding among the evaluated barley accessions, this is to be expected as barley is a self-pollinated crops only 0 to 1.8% outcrossing [55]. Likewise, loci WMC1E8, Ebmac0415, GBM1176 and Bmac0316 exhibited higher level of observed heterozygosity and lower fixation index. This implies that these loci might be associated with inbreeding depression or high mutation rate [56].

The average number of the polymorphic information content (PIC) in the current study was 0.73. [13, 42] reported average PIC values of 0.78 and 0.73 from ICARDA and Ethiopia, respectively. In contrast, lower PIC values of 0.58, 0.54 and 0.46 were reported by [57–59], respectively. In general, a PIC value greater than 0.5 is useful in genetic studies since it can distinguish a marker’s polymorphism [60]. Most of the markers in the current study had a score above 0.5, indicating that they were highly informative SSR markers that could be utilized in research on the genetic diversity of barley. As displayed by diversity parameters, accessions collected from all populations had a relatively high number of effective alleles (3.60) and Shannon’s information index (1.25), indicating a high genetic diversity present in accessions, which may be due to the evolution of cultivated barley [61], topographic variation of the region [5, 20, 23, 39], and the existence of several socio-economic uses and cultural diversity [11]. The study also revealed that accessions collected from Northwest Ethiopia and Central Ethiopia had a higher number of private alleles than grand mean values (0.28), indicating the diversity of this accession and its potential as a source of novel alleles for plant breeding [62].

The genetic distance estimates revealed that a relatively large proportion pairs of accessions (45.10%) had genetic distances higher than the mean Euclidean distance of accessions by one standard deviation. This indicated that relatively many accessions had genetic distance significantly higher than the population mean genetic distance and higher heterosis is expected to be expressed in the crossing made between these pairs of accessions for the traits they had differences. This is because; the accessions had differences for more alleles than alleles possessed by accessions. Generally, twenty (40.81%) accessions had a mean genetic distance of >0.94 (overall mean distances of accessions). The results indicated that at least 20 accessions were distant from other barley accessions by one more allele. The accessions 243191 and 243192 had the highest coefficient of variation of 28.98% followed by 243193 and 243599 with coefficients of variation of 28.96 and 27.02% CV, respectively. The other 13 accessions also had coefficients of variation ranging from 21.99 to 26.83%, which were higher than the average coefficient of variation of the accessions (21.68%) (Table 4). This showed that these accessions had more variation in the alleles than the alleles possessed by the accessions.

The result of AMOVA indicated that variation within population among individuals was higher than the variation among population. The low level of variation among populations collected from four geographic regions indicated that the accessions possessed a high number of common alleles regardless of their geographic origins. This suggested that the higher gene flow across geographic regions and the variation among accessions was more important than looking for differences in geographic regions as a source of genetic distance among accessions to use in barley breeding programs. Other researchers also studied the population structure of barley genotypes and reported that there was more genetic variation among individuals within a population than among populations [13, 37, 63].

Cluster analysis indicates the genetic relationships between the various breeding materials and provides enough information to allow the selection of genetically diverse and superior parental lines for crossing and other breeding programs [64]. The dendrogram of the current study revealed three major clusters and there was no clear separation between accessions based on the geographical locations where they were collected. The present situation confirms that genetic distance is unrelated to geographic distance. Earlier studies on barley revealed comparable results [13, 36, 65, 66].

Cluster I was constructed by accessions collected from northwestern and central Ethiopia. Thus, only accessions obtained from the two geographic regions are included in this cluster; accessions from the other two regions are not included. Cluster II consisted of accessions collected from central, northeastern and southeastern Ethiopia. None of the accessions obtained from northeastern Ethiopia were grouped into subgroups of Clusters I and II. Cluster III was separated into three smaller groups, of which subgroups I, II and III were constructed using accessions collected from southeastern and central Ethiopia, northwestern and northeastern Ethiopia and northeastern and central Ethiopia, respectively. Accessions collected from northeastern Ethiopia were not grouped under subgroup I of Cluster III together with accessions collected from southeastern Ethiopia. Accessions collected from northwestern Ethiopia were grouped into subgroups I and II of Cluster I and subgroup of II of Cluster II along with accessions collected from central Ethiopia.

The remaining seven accessions collected from northwestern Ethiopia were grouped under sub-group II of Cluster III together with accessions collected from northeastern Ethiopia. This showed that the accessions taken from northwestern and northeastern Ethiopia were distant from those collected from southeastern Ethiopia. On the other hand, cluster analysis supports the results shown in AMOVA. This means accessions collected from central Ethiopia were distributed in 6 of the 7 subgroups of the three clusters indicating that the accessions had a close genetic relationship to most of the accessions from the other geographic regions included in this study, and thus variation across accessions is more crucial in determining genetic distance of accessions than looking for variations in regions of origin to use in barley breeding programs This might be due to gene flow, which is facilitated by seed exchange as well as long-distance trading among barley farmers from central Ethiopia with farmers of all geographic regions [37, 66, 67].

Model-based clustering techniques were applied in STRUCTURE software to identify admixed individuals, identify separate genetic groups, and show the existence of population structure [50]. Intermixing distributions of accessions on the principal component axis and admixture structures were observed from the analysis of principal coordinates and population structure, which support the result of the UPGMA dendrogram. The results of the principal coordinate analysis, UPGMA dendrogram, and STRUCTURE analysis proved that there was high gene flow among collection regions, indicating less contribution to genetic variability. The present study showed a certain match with the previous findings [13, 36].

## Conclusion

Research on the genetic diversity of plant species is the most important aspects, of the conservation, improvement, and sustainable utilization of germplasm. This study examined the genetic diversity as well as population structure of 49 barley accessions using 12 polymorphic SSR markers, illustrating an average of 5.08 alleles per locus. Most of the markers were highly informative and efficient for the study of genetic diversity. Accessions from Northwest and Central Ethiopia had a greater number of private alleles, indicating their potential as sources of novel alleles for breeding programs. Genetic distance analysis exhibited significant heterogeneity among accessions. The observed significant genetic variation among the barley accessions will be useful to barley breeders in selecting desirable parents for breeding. Moreover, the Euclidian distance revealed that some accessions obtained from the same geographic regions have a negligible distance. This implies the presence of duplicated accessions conserved in the gene bank and the study provides preliminary information for gene banks to design appropriate conservation techniques. AMOVA demonstrated the presence of high genetic diversity among accessions, compared to the genetic diversity between geographic regions and within accessions. The clustering analysis found three major clusters, with no clear separation based on geographical origin, implying that genetic distance was not related to geographic distance. This underlines the importance of considering genetic variation across accessions rather than focusing on geographical differences in breeding programs. In conclusion, we suggest further investigation by high density SNP marker on a large number of accessions collected from all barley-growing regions and Genome-wide association studies for those accessions having novel alleles.

## Supporting information

S1 Table List and description of barley accessions used in the experiment. (DOCX)

S2 Table Euclidean distances of 49 barley accessions using 12 SSR markers. (XLSX)

## Abbreviations

AMOVA: Analysis of molecular variance
CTAB: Cetyl trimethyl ammonium bromide
DNA: Deoxyribonucleic acid
EBI: Ethiopian Biodiversity Institute
ED: Euclidian distance
He: Expected heterozygosity
Ho: Observed heterozygosity
I: Shannon information index
Na: Number of alleles
Ne: Effective number of alleles
MAF: Major allele frequency
PCoA: Principal coordinate analysis
PCR: Polymerase chain reaction
PIC: Polymorphic information content
PPL: Percentage of polymorphic loci
SSR: Simple sequence repeat
UPGMA: Unweighted pair group method with arithmetic mean
