# Supplementary material for: Analysis of genetic diversity and population structure of some Ethiopian barley (Hordeum vulgare L.) accessions using SSR markers
Source: PLoS One. 2024 Jun 25;19(6):e0305945. doi: 10.1371/journal.pone.0305945 (PMC11198791; doi:10.1371/journal.pone.0305945)
Supplement: S1 Table — (DOCX) [file pone.0305945.s001.docx]

S1 Table. List and description of barley accessions used in the experiment

|  | Accessions | Collection area | | Coordinate | | Altitude (m.a.s.l) |
| --- | --- | --- | --- | --- | --- | --- |
|  |  | Geographical location | Regions | Latitude | Longitude |  |
| 1 | 4423 | North West Ethiopia | Amhara | 10-42-00-N | 37-34-00-E | 2500 |
| 2 | 4425 | North West Ethiopia | Amhara | 10-43-00-N | 37-35-00-E | 2450 |
| 3 | 4426 | North West Ethiopia | Amhara | 10-43-00-N | 37-35-00-E | 2450 |
| 4 | 4427 | North West Ethiopia | Amhara | 10-43-00-N | 37-35-00-E | 2450 |
| 5 | 212737 | North West Ethiopia | Amhara | 37-48-00-N | 10-23-00-E | 2520 |
| 6 | 9950 | North West Ethiopia | Amhara | 10-18-82-N | 37-42-15-E | 2360 |
| 7 | 9949 | North West Ethiopia | Amhara | 10-26-98-N | 37-44-03-E | 2607 |
| 8 | 4366 | North West Ethiopia | Amhara | 10-21-00-N | 37-34-00-E | 2410 |
| 9 | 243286 | North West Ethiopia | Amhara | 11-48-09-N | 38-09-51-E | 2797 |
| 10 | 243287 | North West Ethiopia | Amhara | 11-48-09-N | 38-09-51-E | 2797 |
| 11 | 243288 | North West Ethiopia | Amhara | 11-47-51-N | 38-08-04-E | 2805 |
| 12 | 243289 | North West Ethiopia | Amhara | 11-47-51-N | 38-08-04-E | 2805 |
| 13 | 243597 | North West Ethiopia | Amhara | 13-08-00-N | 37-56-00-E | 3120 |
| 14 | 243598 | North West Ethiopia | Amhara | 13-08-00-N | 37-56-00-E | 3120 |
| 15 | 243599 | North West Ethiopia | Amhara | 13-08-00-N | 37-56-00-E | 3120 |
| 16 | 243600 | North West Ethiopia | Amhara | 13-08-00-N | 37-56-00-E | 3115 |
| 17 | 243568 | North East Ethiopia | Amhara | 11-50-00-N | 39-33-00-E | 2330 |
| 18 | 243571 | North East Ethiopia | Amhara | 11-51-00-N | 39-30-00-E | 2980 |
| 19 | 243572 | North East Ethiopia | Amhara | 11-51-00-N | 39-30-00-E | 2980 |
| 20 | 235066 | North East Ethiopia | Amhara | 11-49-00-N | 39-31-00-E | 3010 |
| 21 | 235072 | North East Ethiopia | Amhara | 11-11-00-N | 39-30-00-E | 2830 |
| 22 | 235073 | North East Ethiopia | Amhara | 11-13-00-N | 39-31-00-E | 2950 |
| 23 | 235074 | North East Ethiopia | Amhara | 11-13-00-N | 39-32-00-E | 2950 |
| 24 | 235075 | North East Ethiopia | Amhara | 11-14-00-N | 39-28-00-E | 3150 |
| 25 | 8525 | Central Ethiopia | Amhara | 09-43-39-N | 39-45-33-E | 2338 |
| 26 | 8526 | Central Ethiopia | Amhara | 09-34-40-N | 39-44-43-E | 2478 |
| 27 | 8556 | Central Ethiopia | Amhara | 09-34-39-N | 39-44-50-E | 2388 |
| 28 | 8557 | Central Ethiopia | Amhara | 09-34-46-N | 39-44-30-E | 2452 |
| 29 | 8558 | Central Ethiopia | Amhara | 09-55-08-N | 39-44-06-E | 2518 |
| 30 | 243232 | Central Ethiopia | Oromia | 09-34-34-N | 39-29-29-E | 2801 |
| 31 | 243231 | Central Ethiopia | Oromia | 09-34-34-N | 39-29-29-E | 2801 |
| 32 | 243230 | Central Ethiopia | Oromia | 09-34-34-N | 39-29-29-E | 2801 |
| 33 | 243229 | Central Ethiopia | Oromia | 09-34-34-N | 39-29-29-E | 2801 |
| 34 | 232219 | Central Ethiopia | Oromia | 08-28-00-N | 39-27-00-E | 2360 |
| 35 | 232220 | Central Ethiopia | Oromia | 08-28-00-N | 39-37-00-E | 2360 |
| 36 | 232221 | Central Ethiopia | Oromia | 08-27-00-N | 39-36-00-E | 2550 |
| 37 | 232222 | Central Ethiopia | Oromia | 08-26-00-N | 39-37-00-E | 2750 |
| 38 | 237002 | Central Ethiopia | Oromia | 07-19-00-N | 39-16-00-E | 2360 |
| 39 | 237003 | Central Ethiopia | Oromia | 07-19-00-N | 39-16-00-E | 2350 |
| 40 | 237004 | Central Ethiopia | Oromia | 07-19-00-N | 39-16-00-E | 2670 |
| 41 | 237011 | Central Ethiopia | Oromia | 07-19-00-N | 39-16-00-E | 2790 |
| 42 | 243191 | South East Ethiopia | Oromia | 07-16-42-N | 39-51-16-E | 2557 |
| 43 | 243192 | South East Ethiopia | Oromia | 07-15-55-N | 39-52-33-E | 2470 |
| 44 | 243193 | South East Ethiopia | Oromia | 07-15-22-N | 39-49-40-E | 2500 |
| 45 | 243195 | South East Ethiopia | Oromia | 07-15-50-N | 39-58-24-E | 2510 |
| 46 | 243213 | South East Ethiopia | Oromia | 07-02-30-N | 39-30-45-E | 2621 |
| 47 | 243214 | South East Ethiopia | Oromia | 07-02-30-N | 39-32-18-E | 2852 |
| 48 | 243215 | South East Ethiopia | Oromia | 07-02-11-N | 39-32-18-E | 2852 |
| 49 | 243216 | South East Ethiopia | Oromia | 07-02-11-N | 39-32-18-E | 2852 |
